# Draft Genome Sequence of Phosphate-Solubilizing Chryseobacterium sp. Strain ISE14, a Biocontrol and Plant Growth-Promoting Rhizobacterium Isolated from Cucumber

**DOI:** 10.1128/genomeA.00612-18

**Published:** 2018-06-28

**Authors:** Jin-Ju Jeong, Mee Kyung Sang, Duleepa Pathiraja, Byeonghyeok Park, In-Geol Choi, Ki Deok Kim

**Affiliations:** a Laboratory of Plant Disease and Biocontrol, Department of Biosystems and Biotechnology, Korea University, Seoul, South Korea; b Division of Agricultural Microbiology, National Academy of Agricultural Science, Rural Development Administration, Wanju, South Korea; c Department of Biotechnology, Korea University, Seoul, South Korea

## Abstract

Chryseobacterium sp. strain ISE14 is a phosphate-solubilizing endophytic bacterium that exhibits plant growth promotion and biocontrol activities against Phytophthora blight and anthracnose on pepper. Here, we report the draft genome sequence of strain ISE14, which contains genes relating to phosphate solubilization, plant growth promotion, and biocontrol traits.

## Abstract (precis)

Chryseobacterium sp. strain ISE14 is a phosphate-solubilizing endophytic bacterium that exhibits plant growth promotion and biocontrol activities against Phytophthora blight and anthracnose on pepper.

## GENOME ANNOUNCEMENT

Bacteria of the genus Chryseobacterium are typically nonmotile, non-spore-forming yellow rods with parallel and rounded shape (1). This genus belonging to the family Flavobacteriaceae was previously considered the genus Flavobacterium; however, in 1994, the genus was distinguished from the genus Flavobacterium (1). Habitats of the free-living or parasitic Chryseobacterium species are chicken, insect gut, plant root, soil, and water (1–6). In our previous studies, Chryseobacterium sp. strain ISE14 exhibited fruit yield increase and biocontrol activity against Phytophthora capsici (causal agent of Phytophthora blight) and Colletotrichum acutatum (causal agent of anthracnose) on pepper plants (7, 8). Recently, Sang et al. (9) demonstrated that strain ISE14 solubilized organic or inorganic phosphate by production of acid and alkaline phosphatases or reduction in pH and promoted pepper plant growth. In addition, this strain was shown to effectively colonize pepper roots. Here, we report the draft genome sequence of the phosphate-solubilizing strain ISE14, which exhibits biocontrol and plant growth promotion activities and was isolated from the surface-sterilized root of a cucumber plant grown in a field in Iksan, South Korea, in 2002 (10).

Genome sequencing of strain ISE14 was conducted using the Illumina MiSeq platform at the Computational and Synthetic Biology Laboratory, Korea University (Seoul, South Korea). A total of 902,913 paired-end reads (103.46-fold coverage) were generated from paired-end sequencing of the genomic library with an average insert size of 500 bp. Low-quality reads were trimmed, with a quality threshold of Q20. The trimmed reads were used for *de novo* assembly using the SPAdes assembler (11). The reads were assembled to 131 scaffolds with a total length of 5,235,650 bp and a G+C content of 36.30%. The maximum length and *N*_50_ value of the contigs were 1,576,022 bp and 1,087,456 bp, respectively. Genome annotation was performed with the Prokaryotic Genome Annotation Pipeline (PGAP) service of NCBI. Among the total of 4,627 complete coding sequences predicted, 4,086 (88.31%) coding sequences showed sequence similarity to known genes in the NCBI database. Additionally, 75 tRNA, 2 5S rRNA, 1 16S rRNA, and 1 23S rRNA gene sequences were retrieved.

The genome analysis of strain ISE14 revealed genes related to colonization ability (e.g., gliding motility lipoprotein) and abiotic or biotic stress tolerance (e.g., glyoxalase, thiamine phosphate synthase, catalase, and peroxidase) (12–15). The genome also contains genes related to antifungal activity (e.g., 3,4-dihydroxy-2-butanone-4-phosphate synthase and flavonol synthase) and plant growth promotion (e.g., production of ammonia and magnesium, phosphate solubilization, and alcohol dehydrogenase) (9, 16, 17). These plant growth-related genes also play important roles in fruit ripening, seedling development, and photosynthesis (18). In conclusion, the genome of Chryseobacterium sp. ISE14 described in this study will be useful to the development of an in-depth understanding of the biocontrol characteristics of the strain, such as antifungal activity, colonization, plant growth promotion, and environmental stress tolerance.

### Accession number(s).

This whole-genome shotgun project has been deposited in the DDBJ/EMBL/GenBank under the accession no. PPED00000000. The version described in this paper is the second version, PPED02000000.
